# Understanding risks of refractive error among Chinese children amidst pandemic disruptions: results from a rapid survey

**DOI:** 10.1186/s12886-021-02133-9

**Published:** 2021-10-19

**Authors:** Ji Liu, Qiaoyi Chen, Jingxia Dang

**Affiliations:** 1 Faculty of Education, Shaanxi Normal University, Xian, Shaanxi China; 2 School of Basic Medical Sciences, Xian Jiaotong University, Xian, Shaanxi China; 3 The First Affiliated Hospital, Xian Jiaotong University, Xian, Shaanxi China

**Keywords:** Eye health, Electronic screens, Psychosocial stress, Outdoor activity, COVID-19

## Abstract

**Background:**

Despite effectiveness in delaying the spread of the pandemic, frequent and extended disruption to children’s livelihoods have fomented new norms in which learning routines encounter immense change. In particular, increased sedentary e-learning engagement with electronic screens and exposure to stressful circumstances are likely to pose adverse risks for children’s vision development.

**Methods:**

This present study examines the link between near-sighted refractive error, and sedentary exposure to electronic screens, psychosocial stress level, and outdoor activities. A Rapid Survey Methodology (RSM) design was utilized to collect information on subject’s vision condition, sedentary electronic screen use, and level of psychosocial stress, in addition to detailed socio-demographic background characteristics.

**Results:**

This study involves 2234 subjects enrolled in 1st to 6th grade in primary schools. Every 1 diopter hour increase in electronic screen use per day is associated with 1.036 OR (95% CI =1.024–1.047, *p*-value< 0.050), while every 1 h • W m^− 2^ sr^− 1^ of illuminance-weighted electronic screen use per day is associated with 2.285 OR (95% CI =1.829–2.855, *p*-value< 0.050) increased likelihood of near-sighted refractive error. Higher level of psychosocial stress is associated with 2.441 OR (95% CI =1.870–3.187, *p*-value< 0.050) and 2.403 OR (95% CI =1.839–3.141, *p*-value< 0.050) increased likelihood of near-sighted refractive error. Frequency of outdoor activity is not significantly associated with increased likelihood of near-sighted refractive error (*p*-value> 0.050).

**Conclusions:**

Findings in this study show that many factors, including grade level and prior vision condition, contribute to increased risks of near-sighted refractive error during the COVID-19 pandemic. More strikingly, pandemic-related behavioral modifications such as lengthy sedentary electronic screen use and elevated levels of psychosocial stress are two critical channels affecting children’s eye health.

## Background

The rapid rise in share of individuals affected by near-sighted refractive error, or myopia, has unfortunately coincided with the COVID-19 global pandemic declared by the World Health Organization (WHO) on March 11th, 2020. As of Aug 1st, 2021, more than 200 million confirmed cases and close to 4.3 million deaths were recorded in more than 200 countries/territories around the world [1]. In response to this ongoing global pandemic, most affected countries/territories have decided to either partially or fully close schools to stop rapid person-to-person disease transmission, which have also impacted the learning of close to 2 billion school-age children worldwide [2].

Globally, a common mitigation strategy to address widespread school-closure policies has been the adoption of remote e-learning engagements, through the use of electronic and telecommunication devices as means of learning at home [3]. While prior research has warned against the probable vision impact of increased remote e-learning arrangements, the degree of these adverse consequences and identification of potential channels through which such risks are realized remain understudied [4–6]. For instance, it is not well-understood how modifications in children’s daily routine and behavior due to extended remote e-learning amidst COVID-19 home confinement, such as increase in sedentary exposure to electronic screens and reduction of outdoor activities, may affect the likelihood of children’s myopic symptom development [7]. In addition, it also remains less clear how risk prevalence of near-sighted refractive error may be heterogeneous across grade levels and location, particularly for children enduring immediate effects of the pandemic [8].

To this end, eye health is important for children’s growth throughout life, and the state of visual acuity at a young age can have significant impact on visual development later in life. Existing studies have suggested that early decline in eye health is associated with elevated risks of developing glaucoma, macular degeneration, and other myopia-related complications that may lead to blindness later in life [9]. In this regard, children’s visual system is extremely delicate, and is in a phase that is characterized by critical development, such that the earlier children become myopic, the more likely they are to develop high myopia and myopia-related vision disorders [10]. Peripheral hyperopic defocus stimulation associated with prolonged electronic screen use can lead to ciliary muscle tension and eye axis elongation, in addition to acute retinal pigment epithelium deterioration due to excessive near-view and high-light exposure [11]. Recent research identifies excessive evaporation of ocular tear fluid during extended visual fixation on electronic visual display terminal as potentially responsible for worsening of eye health [12–15]. At the same time, some in vitro studies highlighted the importance of the integrity of the anterior segment in the visual process [16], while other studies speculate that intensive vitreous chamber lengthening, often due to near-vision high-illuminance, can lead to axial elongation and refractive myopic excursions [17].

To add, acute reduction of outdoor activity can also influence eye health in critical ways, via channels such as the lack of natural light exposure, decrease in depth of visual field, and more frequent eye strain as result of extended indoor near-work and dim-lighting [18]. Moreover, disruption to children’s daily social routine and exposure to stressful living circumstances during the pandemic could also pose adverse risks for children’s mental and physical health [19]. Of particular concern is the rapid symptomatic release of stress hormones due to presence of acute psychosocial stressors, which have been shown to increase propensity of autonomic nervous system malfunction and result in visual field loss and “foggy vision” [20].

In this study, we set out to understand factors associated with risks of developing near-sighted refractive error among young children in China during the COVID-19 pandemic, focusing specifically on their socio-demographic traits and pandemic-led behavioral modifications, including sedentary exposure to electronic screens, psychosocial stress level, and outdoor activities.

## Methods

### Rapid survey design

Between the months of January and May of 2020, all primary schools were closed in China due to the COVID-19 pandemic, and remote e-learning arrangements were made available to all school-age children. In this context, this study utilizes a rapid survey design to capture the state of children’s vision during the first-wave of the COVID-19 pandemic, which was conducted between May 12th to May 18th, 2020 by a nationally-known education press, *Teacher Daily*. Rapid survey methodology (RSM) is a widely adopted data collection approach in developing countries that aims to maximize sample size within a relatively short and fixed timeframe, to allow for quick and timely monitoring of subject’s health status [21].

The study was approved by the Shaanxi Normal University Institutional Review Board, and abided by ethics code of the World Medical Association Declaration of Helsinki. Informed consent for participation in this study was collected from parents/guardians of subjects electronically before commencement of the survey.

Inclusion of subjects in this study were based on the following selection criteria: (1) could fully understand the questionnaire; (2) enrolled in 1st to 6th grade of primary school; (3) fully voluntary participation; (4) recorded only one response under the same IP address. A total of 2234 subjects satisfied the inclusion criteria and are included in the analytic sample.

### Measurements

The survey questionnaire requires approximately 10–15 min to complete, and is intended to guide subjects in reporting demographic background, visual acuity, time use, and stress level during the nationwide school-closures. Importantly, subjects were guided to evaluate occurrence of near-sighted refractive error using the Lay Terms Approach, which anchors on a selection of easy-to-understand and age-appropriate vocabulary to young children, such as “blurry,” “foggy,” “squinting,” etc. [22]. The questionnaire collected detailed information on subjects’ demographic background, daily duration of electronic screen use, type of electronic screen used, daily duration of outdoor activities, and psychosocial stress level ratings.

In this study, we compute proximity-weighted eye use (diopter hours, dh) and illuminance-weighted eye use (h • W m^− 2^ sr^− 1^), leveraging on prior evidence that had evaluated such weighted measures. Specifically, proximity-weighted eye use is computed based on average distance from electronic screens [23], while illuminance-weighted eye use are calculated based on experimental evidence on radiance emitted by different electronic screens [24], using the following formula:

1 $${\displaystyle \begin{array}{l}\mathrm{Proximity}-\mathrm{weighted}\ \mathrm{eye}\ \mathrm{use}=\kern0.5em \left(5\ \mathrm{x}\ \mathrm{dh}\ \mathrm{at}\ 0.2\mathrm{m}\right)\ \\ {}\kern11.75em +\left(2\ \mathrm{x}\ \mathrm{dh}\ \mathrm{at}\ 0.5\mathrm{m}\right)\ \\ {}\kern11.75em +\left(1\ \mathrm{x}\ \mathrm{dh}\ \mathrm{at}\ 1.0\mathrm{m}\right)\end{array}}$$

2 $${\displaystyle \begin{array}{l}\mathrm{Illuminance}-\mathrm{weighted}\ \mathrm{eye}\ \mathrm{use}=\kern0.75em \left(0.26\ \mathrm{h}\bullet \mathrm{W}\ {\mathrm{m}}^{-2}{\mathrm{sr}}^{-1}\mathrm{at}\ 0.2\mathrm{m}\right)\ \\ {}\kern12.75em +\left(0.16\ \mathrm{h}\bullet \mathrm{W}\ {\mathrm{m}}^{-2}{\mathrm{sr}}^{-1}\mathrm{at}\ 0.5\mathrm{m}\right)\\ {}\kern12.75em +\left(0.08\ \mathrm{h}\bullet \mathrm{W}\ {\mathrm{m}}^{-2}{\mathrm{sr}}^{-1}\mathrm{at}\ 1.0\mathrm{m}\right)\end{array}}$$

### Statistical analysis

Statistical analyses were performed using STATA software version 15.0 (Stata, StataCorp LLC, College Station, TX). In this study, we adopt a two-stage analytic plan, where univariate analyses are conducted in the first stage, and multivariate logistic regression models are employed in the second stage. A *p*-value of less than 0.05 was considered to be statistically significant.

Information on sample descriptive statistics, chi-square tests, and paired sample t-test are reported in the first analytic stage in order to evaluate the association between occurrence of near-sighted refractive error (dependent variable) and subjects’ socio-demographic traits. Results from multivariate logistic regression are reported in the second analytic stage as means to assess the risk likelihood of sedentary exposure to electronic screens, psychosocial stress level, and outdoor activities on occurrence of near-sighted refractive error (dependent variable).

## Results

In Table 1, we first present information on sample descriptive statistics, followed by results from chi-square tests and paired sample t-test in the final three columns of the table. Of the 2234 subjects that satisfied the inclusion criteria, more than two-thirds, or 711 (68.2%), reported occurrence of near-sighted refractive error. In the sample, there are 1070 female subjects (47.9%) and 1164 male subjects (52.1%) total. Distribution of subjects across all six grade-levels of primary school fluctuates more or less evenly in a tight range, with 291 (13.0%) in 1st grade, 404 (18.1%) in 2nd grade, 318 (14.2%) in 3rd grade, 420 (18.8%) in 4th grade, 363 (16.2%) in 5th grade, and 438 (19.6%) in 6th grade. In terms of residential location, 453 subjects (20.3%) reside in rural areas, 174 subjects (7.8%) live in urban-rural transitional areas, and 1607 subjects (71.9%) are in urban areas. As for pre-existing myopia condition, 502 subjects (22.5%) report being myopic before start of the COVID-19 pandemic school-closures.

**Table 1:** Sample descriptive statistics and univariate analysis results

Variables	Frequency (% of Total)	Occurrence of Near-sighted Refractive Error (Yes = 1, No = 0)		
		Frequency (Yes = 1)	% of Category	*p*-value
Occurrence of Near-sighted Refractive Error				
Yes	711 (68.2)	–	–	–
No	1523 (31.8)	–	–	–
Sex ^a^				
Female	1070 (47.9)	338	31.6	0.817
Male	1164 (52.1)	373	32.0	
Grade Level ^a^				
1st	291 (13.0)	73	25.1	0.061
2nd	404 (18.1)	120	29.7	
3rd	318 (14.2)	109	34.3	
4th	420 (18.8)	133	31.7	
5th	363 (16.2)	128	35.3	
6th	438 (19.6)	148	33.8	
Residential Location ^a^				
Rural	453 (20.3)	152	33.6	0.289
Urban-Rural Transitional	174 (7.8)	47	27.0	
Urban	1607 (71.9)	512	31.9	
Prior Refractive Error Condition ^a^				
Yes	502 (22.5)	286	57.0	0.000***
No	1732 (77.5)	425	24.5	
Proximity-weighted e-Screen Use, dh (mean, s.d.) ^b^	11.04 (8.91)	mean (1) – mean (0) = 3.64		0.000***
Illuminance-weighted e-Screen Use, h • W m^−2^ sr ^−1^ (mean, s.d.) ^b^	0.64 (0.45)	mean (1) – mean (0) = 0.21		0.000***
Level of Psychosocial Stress ^a^				
Apprehensive	318 (14.2)	176	55.4	0.000***
Composed	375 (16.8)	75	20.0	
Indifferent	1541 (69.0)	460	29.9	
Frequency of Outdoor Activity ^a^				
7 times/week	414 (18.5)	135	32.6	0.088
4–6 times/week	288 (12.9)	79	27.4	
1–3 times/week	1123 (50.3)	349	31.1	
0 times/week	409 (18.3)	148	36.2	

^a^ *p*-value based on χ^2^ test, ^b^ *p*-value based on T-test. *** indicates *p*-value< 0.050

Information on sedentary exposure to electronic screens is reported daily and in terms of proximity-weighted diopter hours and illuminance-weighted hours. On average, subjects in the sample indicate using electronic screens 11.04 dh per day (SD = 8.91) and 0.64 h • W m^− 2^ sr^− 1^ per day (SD = 0.45). For assessment of psychosocial stress, 318 subjects (14.2%) reported as being “Apprehensive,” 375 subjects (16.8%) reported as being “Composed,” while 1541 subjects (69.0%) indicated as “Indifferent.” For frequency of outdoor activity, 414 subjects (18.5%) report 7 times per week, 288 subjects (12.9%) report between 4 and 6 times per week, 1123 (50.3%) report between 1 and 3 times per week, while 409 subjects report 0 times per week.

In terms of univariate association between subjects’ occurrence of near-sighted refractive error and the above variables, several observations can be made. First, there is no statistically significant relationship between occurrence of near-sighted refractive error and sex (χ^2^ = 0.053, *p*-value> 0.050), grade (χ^2^ = 10.570, *p*-value> 0.050), residential location (χ^2^ = 2.483, *p*-value> 0.050), and frequency of outdoor activity (χ^2^ = 6.554, *p*-value> 0.050). Second, those subjects reporting refractive error condition before start of the COVID-19 pandemic school-closures are more likely to also experience occurrence of near-sighted refractive error during the COVID-19 school closures (χ^2^ = 188.678, *p*-value< 0.050). Third, subjects reporting occurrence of near-sighted refractive error on average indicate 3.64 dh (t-statistic =9.161, *p*-value< 0.050) and 0.21 h • W m^− 2^ sr^− 1^ (t-statistic =10.337, *p*-value< 0.050) additional exposure to electronic screens per day than subjects who did not experience near-sighted refractive error. Fourth, subjects who report being under “Stressful” circumstances also indicate occurrence of near-sighted refractive error at higher proportions (χ^2^ = 108.019, *p*-value< 0.050).

Utilizing two separate multivariate logistic regression models, with the first using proximity-weighted measure of electronic screen exposure and the second based on illuminance-weighted measure of electronic screen exposure, Table 2 presents detailed results assessing the risk likelihood of exposure to electronic screens, psychosocial stress level, and outdoor activities on occurrence of near-sighted refractive error (dependent variable), while holding socio-demographic variable constant. This differentiation in statistical modelling allows for further discussion on the channels through which adverse vision risks are realized, whether through close-proximity of e-screen use or through high-light emission exposure.

**Table 2:** Multivariate logistical regression analysis results

Variables	DV: Occurrence of Near-sighted Refractive Error (Yes = 1, No = 0)					
	Model 1 (Proximity-weighted, dh)			Model 2 (Illuminance-weighted, h•W m^−2^ sr^−1^)		
	OR	95% CI	*p*-value	OR	95% CI	*p*-value
e-Screen Use, daily hours	1.036	1.024–1.047	0.000***	2.285	1.829–2.855	0.000***
Level of Psychosocial Stress						
Apprehensive	2.441	1.870–3.187	0.000***	2.403	1.839–3.141	0.000***
Composed	0.671	0.503–0.894	0.006***	0.667	0.500–0.890	0.006***
Indifferent	1			1		
Frequency Outdoor Activity						
7 times/week	1.189	0.866–1.632	0.284	1.222	0.889–1.681	0.217
4–6 times/week	0.871	0.610–1.244	0.447	0.881	0.616–1.261	0.489
1–3 times/week	0.968	0.745–1.258	0.809	0.972	0.747–1.265	0.831
0 times/week	1			1		
Sex						
Female	0.960	0.791–1.164	0.675	0.957	0.788–1.162	0.658
Male	1			1		
Grade Level						
1st	1.335	1.044–1.930	0.018***	1.428	1.080–2.071	0.030***
2nd	1.455	1.047–2.022	0.025***	1.522	1.092–2.122	0.013***
3rd	1.469	1.042–2.069	0.028***	1.474	1.045–2.079	0.027***
4th	1.289	0.939–1.769	0.116	1.334	0.970–1.834	0.076
5th	1.178	0.852–1.629	0.323	1.171	0.846–1.622	0.340
6th	1			1		
Residential Location						
Rural	1.036	0.808–1.326	0.782	1.020	0.796–1.307	0.876
Urban-Rural	0.767	0.523–1.125	0.175	0.764	0.520–1.123	0.171
Urban	1			1		
Prior Refractive Error Condition						
Yes	3.853	3.077–4.826	0.000***	3.827	3.053–4.798	0.000***
No	1			1		

*OR* odds ratio, *CI* confidence interval

*** indicates *p*-value< 0.050

Across both Models 1 and 2, results show that e-screen use is positively associated with elevated risks of near-sighted refractive error. On the one hand, each additional diopter hour increase in electronic screen use per day is associated with 1.036 odds ratio (OR) (95% Confidence Interval, CI =1.024–1.047, *p*-value< 0.050) increased likelihood of near-sighted refractive error. On the other hand, every 1 h • W m^− 2^ sr^− 1^ of illuminance-weighted electronic screen use per day is associated with 2.285 OR (95% CI =1.829–2.855, *p*-value< 0.050) increased propensity in near-sighted refractive error.

In addition, across both Models 1 and 2, “Apprehensive” level of psychosocial stress is associated with elevated risks in occurrence of near-sighted refractive error, such that Model 1 predicts 2.441 OR (95% CI =1.870–3.187, *p*-value< 0.050) and Model 2 predicts 2.403 OR (95% CI =1.839–3.141, *p*-value< 0.050). Conversely, “Composed” level of psychosocial stress is associated with decreased risks in occurrence of near-sighted refractive error, with Model 1 predicting 0.671 OR (95% CI =0.503–0.894, *p*-value< 0.050) and Model 2 estimating 0.667 OR (95% CI =0.500–0.890, *p*-value< 0.050).

Moreover, in both Models 1 and 2, results suggest that lower grade-levels are shown to exhibit higher risks of developing near-sighted refractive error. For instance, Model 1 estimates that odds ratios of 1st grade, 2nd grade, 3rd grade in relation to 6th grade are 1.335 OR (95% CI =1.044–1.930, *p*-value< 0.050), 1.455 OR (95% CI =1.047–2.022, *p*-value< 0.050), and 1.469 OR (95% CI =1.042–2.069, *p*-value< 0.050) respectively. Results are statistically similar in Model 2, such that the odds ratios are 1.428 OR (95% CI =1.080–2.071, *p*-value< 0.050), 1.522 OR (95% CI =1.092–2.122, *p*-value< 0.050), and 1.474 OR (95% CI =1.045–2.079, *p*-value< 0.050), respectively. Additionally, risks of near-sighted refractive error are also categorically higher for subjects reporting refractive error condition before start of the COVID-19 pandemic school-closures, evaluated at 3.853 OR (95% CI =3.077–4.826, *p*-value< 0.050) and 3.827 OR (95% CI =3.053–4.798, *p*-value< 0.050) for Models 1 and 2, respectively.

Noteworthy, both Models 1 and 2 concurrently predict that frequency of outdoor activity is not significantly associated with occurrence of near-sighted refractive error (*p*-value> 0.050), and so are the relationships for sex (*p*-value> 0.050) and residential location (*p*-value> 0.050).

## Discussion

This study investigated a series of factors that could be associated with risks of developing myopic refractive error among young children in China during the COVID-19 pandemic, focusing on children’s behavioral modifications, including proximity-weighted and illuminance-weighted exposure to electronic screens, levels of psychosocial stress, and frequency of engagement in outdoor activities. School-age children were recruited utilizing an RSM design, and a total of 2234 subjects satisfied the inclusion criteria to form the analytic sample in this study.

Results from the survey reveal that subjects, on average, use electronic screens 11.04 dh per day (SD = 8.91) and 0.64 h • W m^− 2^ sr^− 1^ per day (SD = 0.45). According to World Health Organization eye health guidelines, daily use of digital electronic screens of more than 2 h is not recommended for young children [25]. More worryingly, univariate analyses show that both proximity-weighted and illuminance-weighted measures of electronic screen use are associated with higher likelihood of near-sighted refractive error occurrence among subjects (*p*-value< 0.050). Using multiple logistic regression, point estimates indicate that each additional diopter hour is associated with 1.036 OR (95% CI =1.024–1.047, *p*-value< 0.050) and every 1 h • W m^− 2^ sr^− 1^ is associated with 2.285 OR (95% CI =1.829–2.855, *p*-value< 0.050), increased likelihood of near-sighted refractive error. These results translate into sizable eye health risks considering that subjects report 11.04 dh and 0.64 h • W m^− 2^ sr^− 1^ per day on average, and highlights close-proximity of e-screen use as a main source of vision risk affecting children’s eye health.

Notably, these findings are consistent with emerging evidence that changes in behavior norms during the pandemic have created favorable myopigenic conditions which can have consequential adverse effects on eye health among school-age children [26, 27]. In consideration of the longer-term repercussions stemming from these results, governments, schools, and families should come together to make collective efforts in prevention of myopigenic behavioral changes, such as issuing electronic screen viewing distance recommendation and duration of device use reminder interventions, particularly during times of the COVID-19 pandemic [28].

In addition, this study also evaluated the extent to which psychosocial stress level and frequency of outdoor activity is related to occurrence of near-sighted refractive error. Particularly, heightened level of psychosocial stress is positively correlated with increased risk of developing myopic refractive error (*p*-value< 0.050), while frequency of outdoor activity does not seem to correlate with elevated risks of refractive error (*p*-value> 0.050). On the one hand, these findings are in line with recent investigations in confirming that children’s state of mental health during the pandemic can have influential effects on their physical health [29], while contributing new evidence on the link between psychosocial stress and eye health among young children. On the other hand, this study engages existing literature debating the association between outdoor activity and myopic refractive error in children [30], and illustrate that this link may be quite complex during COVID-19 [31], because variation in outdoor activities is substantially minimized due to extensive pandemic-related public curfew measures.

Nevertheless, there are a few limitations in this study that needs to be accounted for in future research. First, this study elected an RSM design in consideration of the time-sensitive, logistical, and public health circumstances that are unique to the COVID-19 pandemic. However, studies utilizing RSM are prone to nonrandom sampling bias due to the voluntary nature of study enrollment, which is nevertheless not entirely uncommon in other observational studies. Second, this study prompted subjects to evaluate vision status using the Lay Terms approach, instead of relying on eye specialist examinations. While the Lay Terms approach has been previously shown not to differ systematically from ophthalmologist assessments [20], future studies may consider the inclusion of eye specialist examinations for data validation and triangulation purposes, should social-distancing requirements become lifted.

In conclusion, findings in this study show that the scale at which COVID-19 is affecting children’s lives, behaviors, and vision development is unprecedented. While some of these consequences are less noticeable than others, their long-term effects hold important weight in determining the status of children’s health and wellbeing for an entire generation to come, intervening in their vision growth during a critical yet delicate phase. In light of several education systems continuing to rely on remote distance learning modalities in on-going and future waves of the pandemic, findings in this study highlight the urgency of developing appropriate mitigation strategies to address the increasing likelihood of a global myopia crisis.

## Data Availability

Restrictions apply to the availability of data used, due to study subject privacy protection. Deidentified participant data is available upon reasonable request to the corresponding author, with permission of Teachers Daily.
